# Characterisation of plasma phosphorylated Tau181 in a middle‐aged birth cohort: Associations with cognitive decline, brain structural integrity, and accelerated ageing

**DOI:** 10.1002/alz70856_099207

**Published:** 2025-12-24

**Authors:** Ashleigh Barrett‐Young, Erin E Cawston, Brigid Ryan, Wickliffe C. Abraham, Antony Ambler, Tim J Anderson, Kirsten Cheyne, Elizabeth Goodin, Sean Hogan, Renate Houts, David Ireland, Annchen R. Knodt, Jesse Kokaua, Tracy R Melzer, Sandhya Ramrakha, Karen Sugden, Benjamin Williams, Phillipa Wilson, Avshalom Caspi, Ahmad R. Hariri, Terrie E. Moffitt, Richie Poulton, Reremoana Theodore

**Affiliations:** ^1^ University of Otago, Dunedin, Otago, New Zealand; ^2^ University of Auckland, Auckland, Auckland, New Zealand; ^3^ University of Auckland, Auckland, New Zealand; ^4^ Department of Psychology, Brain Health Research Centre, Brain Research New Zealand, University of Otago, Dunedin, New Zealand; ^5^ University of Otago, Christchurch, Canterbury, New Zealand; ^6^ Duke University, Durham, NC, USA; ^7^ New Zealand Brain Research Institute, Christchurch, New Zealand; ^8^ University of Otago, Dunedin, New Zealand; ^9^ King's College London, London, United Kingdom

## Abstract

**Background:**

Although plasma pTau181 has been shown to accurately identify patients with AD in case‐control studies, its utility as a preclinical biomarker in middle‐aged community‐based cohorts is unclear. Our objective was to investigate whether plasma pTau181 was associated with indicators of AD risk in a middle‐aged cohort (aged 45 years).

**Method:**

Participants were members of the Dunedin Multidisciplinary Health and Development Study, a longitudinal study of 1037 people born in New Zealand in 1972‐1973. Most recently, 94% of the living Study members were assessed at age 45 (2017‐2019). Plasma pTau181 was measured using the Simoa SR‐X analyser (Quanterix). MRI scans were conducted at the same timepoint. Cognitive ability was assessed at ages 7, 9, 11, and at age 45. Pace of ageing, a composite measure of 19 biomarkers of ageing, was determined from data collected at ages 32, 38, and 45.

**Result:**

We observed a wide range of pTau181 levels in our same‐aged sample (*n* = 856), from below the lower limit of detection to 147pg/ml (M=13.6pg/ml, SD=9.1). Excluding n<5 participants with chronic kidney disease, males had significantly higher pTau181 levels than females. No statistically significant associations were observed with cognitive measures, MRI‐derived brain measurements, or composite ageing variables.

**Conclusion:**

The range of pTau181 levels observed suggests that blood biomarkers of AD may increase early in middle age, decades prior to any potential disease onset and possibly before any measurable cognitive decline or neurostructural abnormalities. Future assessment waves in the same cohort will elucidate whether plasma pTau181 in midlife is predictive of AD onset later in life.

